# (2*R*,3a*R*,4*S*,7*R*,7a*S*,9*R*,10a*R*,11*S*,14*R*,14a*S*)-*rel*-3a,4,7,7a,10a,11,14,14a-Octa­hydro-4,14:7,11-diep­oxy-2,9-propanona­phtho[1,2-*f*:5,6-*f*′]diisoindole-1,3,8,10-tetrone (9CI): a cyclo­phane derived from naphtho[1,2-*c*:5,6-*c*]difuran

**DOI:** 10.1107/S1600536808025397

**Published:** 2008-08-30

**Authors:** Michelle E. Thibault, Masood Parvez, Peter W. Dibble

**Affiliations:** a Department of Chemistry and Biochemistry, University of Lethbridge, Lethbridge, Alberta, Canada T1K 3M4; b Department of Chemistry, The University of Calgary, 2500 University Drive NW, Calgary, Alberta, Canada T2N 1N4

## Abstract

The title compound, C_25_H_18_N_2_O_6_, is a naphthalenophane styled in the manner of Warrener’s alicyclic cyclo­phanes or mol­ecular racks wherein a trimethyl­ene tether is perfectly staggered between the two N atoms such that the central methyl­ene H atoms point toward the naphthalene π-system. The dihedral angle between the mean planes of the two benzene rings is 7.61 (7)°.

## Related literature

For related literature, see: Butler *et al.* (2000 ▶); Thibault *et al.* (2003 ▶).
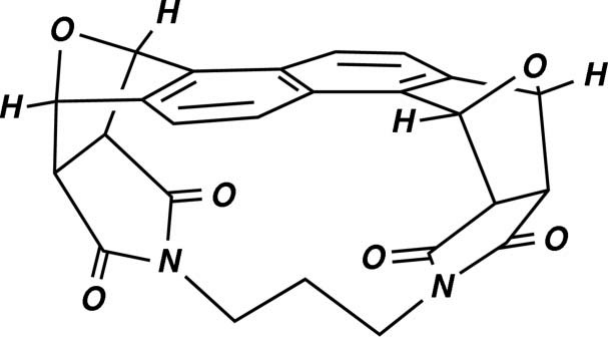


## Experimental

### 

#### Crystal data

C_25_H_18_N_2_O_6_
*M* *_r_* = 442.41
Tetragonal, 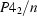

*a* = 21.635 (9) Å
*c* = 8.262 (2) Å
*V* = 3867 (2) Å^3^
*Z* = 8
Mo *K*α radiation
μ = 0.11 mm^−1^
*T* = 173 (2) K
0.35 × 0.12 × 0.12 mm

#### Data collection

Nonius KappaCCD diffractometer
Absorption correction: multi-scan (*SORTAV*; Blessing, 1997 ▶) *T*_min_ = 0.962, *T*_max_ = 0.987
7455 measured reflections
4303 independent reflections
3077 reflections with *I* > 2σ(*I*)
*R*_int_ = 0.030

#### Refinement

*R*[*F* ^2^ > 2σ(*F* ^2^)] = 0.041
*wR*(*F* ^2^) = 0.107
*S* = 1.02
4303 reflections
298 parameters
H-atom parameters constrained
Δρ_max_ = 0.19 e Å^−3^
Δρ_min_ = −0.18 e Å^−3^

### 

Data collection: *COLLECT* (Hooft, 1998 ▶); cell refinement: *DENZO* (Otwinowski & Minor, 1997 ▶); data reduction: *SCALEPACK* (Otwinowski & Minor, 1997 ▶); program(s) used to solve structure: *SAPI91* (Fan, 1991 ▶); program(s) used to refine structure: *SHELXL97* (Sheldrick, 2008 ▶); molecular graphics: *ORTEP-3 for Windows* (Farrugia, 1997 ▶); software used to prepare material for publication: *SHELXL97*.

## Supplementary Material

Crystal structure: contains datablocks global, I. DOI: 10.1107/S1600536808025397/lh2675sup1.cif

Structure factors: contains datablocks I. DOI: 10.1107/S1600536808025397/lh2675Isup2.hkl

Additional supplementary materials:  crystallographic information; 3D view; checkCIF report

## Figures and Tables

**Fig. 1.**
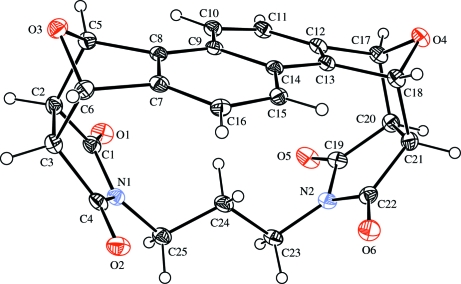
ORTEP-3 (Farrugia, 1997) drawing of the title compound with displacement ellipsoids plotted at 50% probability level.

**Fig. 2.**
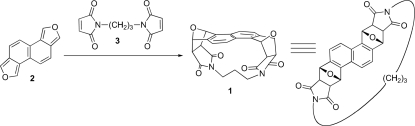
The formation of the title compound.

## supplementary crystallographic information

### Comment

The title compound, (1), was prepared by the Diels-Alder reaction of naphtho[1,2 - c:5,6 - c]difuran (2) and 1,3-bis(maleimido)propane (3) (see Fig. 2). It is a naphthalenophane styled in the manner of Warreners alicyclic cyclophanes or molecular racks. (Butler *et al.*, 2000) The X-ray structure shows that the three carbon methylene tether is perfectly staggered between the two N atoms such that the central methylene protons point toward the naphthalenic π-system. There is a slight 'warp' to the naphthalene rings system with a mean-planes angle between the two benzene rings being 7.61 (7)°. The only anomalous bond angle in the molecule appears in the methylene tether with the central angle (C23—C24—C25) expanded to 115.17 (13)°, evidence of a repulsive interaction with the naphthalene ring.

### Experimental

The preparation of the title compound has been reported (Thibault *et al.*, 2003).

### Refinement

Though all the H atoms could be distinguished in the difference Fourier map the H-atoms were included at geometrically idealized positions and refined in riding-model approximation with the C—H distances set in the range 0.95 - 1.00 Å, and *U*_iso_(H) = 1.2 *U*_eq_(C). The final difference map was free of any chemically significant features.

### Crystal data

**Table:**

C_25_H_18_N_2_O_6_	*Z* = 8
*M**_r_* = 442.41	*F*_000_ = 1840
Tetragonal, *P*4_2_/*n*	*D*_x_ = 1.520 Mg m^−^^3^
Hall symbol: -P 4bc	Mo *K*α radiation λ = 0.71073 Å
*a* = 21.635 (9) Å	Cell parameters from 3862 reflections
*b* = 21.635 (9) Å	θ = 1.0–27.5º
*c* = 8.262 (2) Å	µ = 0.11 mm^−^^1^
α = 90º	*T* = 173 (2) K
β = 90º	Needle, colorless
γ = 90º	0.35 × 0.12 × 0.12 mm
*V* = 3867 (2) Å^3^	

### Data collection

**Table:**

Nonius KappaCCD diffractometer	4303 independent reflections
Radiation source: fine-focus sealed tube	3077 reflections with *I* > 2σ(*I*)
Monochromator: graphite	*R*_int_ = 0.030
*T* = 173(2) K	θ_max_ = 27.5º
ω and φ scans	θ_min_ = 1.3º
Absorption correction: multi-scan(SORTAV; Blessing, 1997)	*h* = −27→28
*T*_min_ = 0.962, *T*_max_ = 0.987	*k* = −19→19
7455 measured reflections	*l* = −9→10

### Refinement

**Table:**

Refinement on *F*^2^	Secondary atom site location: difference Fourier map
Least-squares matrix: full	Hydrogen site location: inferred from neighbouring sites
*R*[*F*^2^ > 2σ(*F*^2^)] = 0.041	H-atom parameters constrained
*wR*(*F*^2^) = 0.107	*w* = 1/[σ^2^(*F*_o_^2^) + (0.0461*P*)^2^ + 0.9086*P*] where *P* = (*F*_o_^2^ + 2*F*_c_^2^)/3
*S* = 1.02	(Δ/σ)_max_ < 0.001
4303 reflections	Δρ_max_ = 0.19 e Å^−^^3^
298 parameters	Δρ_min_ = −0.18 e Å^−^^3^
Primary atom site location: structure-invariant direct methods	Extinction correction: none

### Special details

**Table:**

Geometry. All e.s.d.'s (except the e.s.d. in the dihedral angle between two l.s. planes) are estimated using the full covariance matrix. The cell e.s.d.'s are taken into account individually in the estimation of e.s.d.'s in distances, angles and torsion angles; correlations between e.s.d.'s in cell parameters are only used when they are defined by crystal symmetry. An approximate (isotropic) treatment of cell e.s.d.'s is used for estimating e.s.d.'s involving l.s. planes.
Refinement. Refinement of *F*^2^ against ALL reflections. The weighted *R*-factor *wR* and goodness of fit *S* are based on *F*^2^, conventional *R*-factors *R* are based on *F*, with *F* set to zero for negative *F*^2^. The threshold expression of *F*^2^ > σ(*F*^2^) is used only for calculating *R*-factors(gt) *etc*. and is not relevant to the choice of reflections for refinement. *R*-factors based on *F*^2^ are statistically about twice as large as those based on *F*, and *R*- factors based on ALL data will be even larger.

### Fractional atomic coordinates and isotropic or equivalent isotropic displacement parameters (Å^2^)

**Table:**

	*x*	*y*	*z*	*U*_iso_*/*U*_eq_	
O1	0.22246 (5)	0.13740 (6)	0.23245 (15)	0.0336 (3)	
O2	0.01829 (5)	0.13616 (6)	0.36524 (14)	0.0309 (3)	
O3	0.16200 (5)	0.15123 (5)	0.75206 (13)	0.0272 (3)	
O4	0.19993 (5)	−0.19809 (5)	0.40461 (14)	0.0273 (3)	
O5	0.22497 (6)	−0.07494 (6)	−0.02726 (16)	0.0399 (3)	
O6	0.03367 (5)	−0.10018 (6)	0.19059 (15)	0.0328 (3)	
N1	0.11727 (6)	0.13315 (6)	0.26487 (16)	0.0227 (3)	
N2	0.12520 (6)	−0.07665 (6)	0.06537 (16)	0.0244 (3)	
C1	0.17687 (7)	0.14753 (7)	0.3135 (2)	0.0244 (4)	
C2	0.17425 (7)	0.17364 (7)	0.4820 (2)	0.0235 (4)	
H2	0.1943	0.2152	0.4882	0.028*	
C3	0.10474 (7)	0.17687 (7)	0.52369 (19)	0.0236 (4)	
H3	0.0907	0.2203	0.5420	0.028*	
C4	0.07282 (7)	0.14768 (7)	0.38054 (19)	0.0232 (4)	
C5	0.19873 (8)	0.12925 (7)	0.6167 (2)	0.0241 (4)	
H5	0.2445	0.1302	0.6339	0.029*	
C6	0.10185 (7)	0.13748 (7)	0.68267 (19)	0.0240 (4)	
H6	0.0657	0.1462	0.7546	0.029*	
C7	0.11018 (7)	0.07033 (7)	0.63576 (18)	0.0214 (3)	
C8	0.17119 (7)	0.06510 (7)	0.59077 (18)	0.0205 (3)	
C9	0.19457 (7)	0.01012 (7)	0.52115 (18)	0.0202 (3)	
C10	0.25447 (7)	0.00473 (8)	0.45215 (19)	0.0241 (4)	
H10	0.2825	0.0384	0.4612	0.029*	
C11	0.27291 (7)	−0.04800 (8)	0.3725 (2)	0.0254 (4)	
H11	0.3122	−0.0502	0.3214	0.030*	
C12	0.23214 (7)	−0.09846 (7)	0.36885 (19)	0.0232 (4)	
C13	0.17471 (7)	−0.09597 (7)	0.44037 (19)	0.0208 (3)	
C14	0.15216 (7)	−0.04141 (7)	0.51354 (18)	0.0195 (3)	
C15	0.09034 (7)	−0.03480 (7)	0.56703 (18)	0.0212 (3)	
H15	0.0635	−0.0695	0.5644	0.025*	
C16	0.06846 (7)	0.02078 (7)	0.62255 (18)	0.0215 (3)	
H16	0.0262	0.0257	0.6513	0.026*	
C17	0.23450 (8)	−0.16132 (8)	0.2891 (2)	0.0257 (4)	
H17	0.2769	−0.1769	0.2637	0.031*	
C18	0.14564 (7)	−0.15878 (7)	0.4099 (2)	0.0244 (4)	
H18	0.1137	−0.1714	0.4910	0.029*	
C19	0.18458 (8)	−0.10055 (8)	0.04906 (19)	0.0268 (4)	
C20	0.18801 (7)	−0.16089 (8)	0.1424 (2)	0.0263 (4)	
H20	0.1949	−0.1969	0.0688	0.032*	
C21	0.12505 (8)	−0.16503 (8)	0.2289 (2)	0.0252 (4)	
H21	0.1045	−0.2056	0.2082	0.030*	
C22	0.08744 (8)	−0.11216 (8)	0.16359 (19)	0.0246 (4)	
C23	0.10842 (8)	−0.01442 (7)	0.01244 (19)	0.0260 (4)	
H23A	0.0646	−0.0134	−0.0219	0.031*	
H23B	0.1344	−0.0019	−0.0805	0.031*	
C24	0.11844 (8)	0.02956 (8)	0.1539 (2)	0.0275 (4)	
H24A	0.0920	0.0160	0.2451	0.033*	
H24B	0.1620	0.0261	0.1896	0.033*	
C25	0.10459 (8)	0.09703 (8)	0.1186 (2)	0.0266 (4)	
H25A	0.1308	0.1118	0.0284	0.032*	
H25B	0.0607	0.1019	0.0868	0.032*	

### Atomic displacement parameters (Å^2^)

**Table:**

	*U*^11^	*U*^22^	*U*^33^	*U*^12^	*U*^13^	*U*^23^
O1	0.0261 (7)	0.0423 (8)	0.0323 (7)	0.0000 (5)	0.0082 (5)	0.0044 (5)
O2	0.0228 (7)	0.0364 (7)	0.0334 (7)	−0.0002 (5)	−0.0026 (5)	0.0081 (5)
O3	0.0297 (7)	0.0280 (7)	0.0240 (6)	−0.0019 (5)	−0.0018 (5)	−0.0028 (5)
O4	0.0274 (7)	0.0232 (6)	0.0313 (7)	0.0046 (5)	0.0033 (5)	0.0054 (5)
O5	0.0330 (7)	0.0468 (8)	0.0399 (8)	0.0089 (6)	0.0131 (6)	0.0141 (6)
O6	0.0237 (7)	0.0353 (7)	0.0394 (7)	0.0039 (5)	0.0034 (5)	−0.0018 (5)
N1	0.0226 (7)	0.0226 (7)	0.0229 (7)	0.0004 (6)	−0.0001 (5)	0.0039 (5)
N2	0.0247 (7)	0.0271 (8)	0.0214 (7)	0.0053 (6)	0.0007 (5)	0.0001 (6)
C1	0.0235 (9)	0.0224 (9)	0.0273 (9)	−0.0010 (7)	0.0014 (7)	0.0081 (7)
C2	0.0228 (9)	0.0205 (8)	0.0273 (9)	−0.0022 (6)	0.0000 (6)	0.0021 (6)
C3	0.0245 (9)	0.0199 (9)	0.0264 (9)	0.0010 (6)	0.0012 (6)	0.0019 (6)
C4	0.0226 (9)	0.0202 (8)	0.0267 (9)	0.0029 (7)	0.0002 (7)	0.0093 (6)
C5	0.0227 (9)	0.0241 (9)	0.0254 (9)	−0.0003 (7)	−0.0014 (7)	0.0003 (6)
C6	0.0225 (9)	0.0264 (9)	0.0231 (8)	−0.0014 (7)	0.0003 (6)	−0.0007 (7)
C7	0.0255 (9)	0.0248 (9)	0.0140 (8)	0.0013 (7)	0.0000 (6)	0.0035 (6)
C8	0.0224 (8)	0.0213 (8)	0.0178 (8)	−0.0006 (6)	−0.0035 (6)	0.0041 (6)
C9	0.0194 (8)	0.0229 (8)	0.0184 (8)	0.0006 (6)	−0.0032 (6)	0.0050 (6)
C10	0.0183 (8)	0.0265 (9)	0.0275 (9)	−0.0018 (7)	−0.0021 (6)	0.0059 (7)
C11	0.0189 (9)	0.0288 (9)	0.0284 (9)	0.0017 (7)	0.0017 (6)	0.0063 (7)
C12	0.0238 (9)	0.0241 (9)	0.0216 (8)	0.0042 (7)	0.0001 (6)	0.0052 (6)
C13	0.0210 (8)	0.0216 (8)	0.0197 (8)	0.0010 (6)	−0.0009 (6)	0.0036 (6)
C14	0.0213 (8)	0.0216 (8)	0.0156 (8)	−0.0006 (6)	−0.0019 (6)	0.0049 (6)
C15	0.0223 (8)	0.0234 (9)	0.0179 (8)	−0.0034 (7)	0.0000 (6)	0.0039 (6)
C16	0.0199 (8)	0.0272 (9)	0.0174 (8)	−0.0007 (6)	0.0009 (6)	0.0029 (6)
C17	0.0226 (9)	0.0268 (9)	0.0277 (9)	0.0060 (7)	0.0037 (7)	0.0036 (7)
C18	0.0249 (9)	0.0232 (9)	0.0251 (9)	0.0020 (7)	0.0024 (7)	0.0027 (6)
C19	0.0261 (9)	0.0330 (10)	0.0213 (8)	0.0059 (7)	0.0030 (7)	−0.0013 (7)
C20	0.0274 (9)	0.0250 (9)	0.0266 (9)	0.0041 (7)	0.0028 (7)	−0.0028 (7)
C21	0.0264 (9)	0.0224 (9)	0.0268 (9)	0.0008 (7)	0.0002 (7)	−0.0030 (7)
C22	0.0248 (9)	0.0269 (9)	0.0220 (8)	0.0010 (7)	−0.0005 (7)	−0.0066 (6)
C23	0.0280 (9)	0.0288 (9)	0.0213 (8)	0.0062 (7)	−0.0018 (7)	0.0034 (7)
C24	0.0339 (10)	0.0275 (9)	0.0210 (9)	0.0040 (7)	−0.0036 (7)	0.0035 (7)
C25	0.0305 (9)	0.0278 (9)	0.0214 (9)	0.0023 (7)	−0.0016 (7)	0.0040 (7)

### Geometric parameters (Å, °)

**Table:**

O1—C1	1.212 (2)	C10—C11	1.376 (2)
O2—C4	1.212 (2)	C10—H10	0.9500
O3—C5	1.452 (2)	C11—C12	1.404 (2)
O3—C6	1.453 (2)	C11—H11	0.9500
O4—C18	1.451 (2)	C12—C13	1.377 (2)
O4—C17	1.450 (2)	C12—C17	1.512 (2)
O5—C19	1.212 (2)	C13—C14	1.413 (2)
O6—C22	1.213 (2)	C13—C18	1.518 (2)
N1—C1	1.386 (2)	C14—C15	1.416 (2)
N1—C4	1.392 (2)	C15—C16	1.371 (2)
N1—C25	1.465 (2)	C15—H15	0.9500
N2—C22	1.384 (2)	C16—H16	0.9500
N2—C19	1.391 (2)	C17—C20	1.575 (2)
N2—C23	1.461 (2)	C17—H17	1.0000
C1—C2	1.503 (2)	C18—C21	1.567 (2)
C2—C3	1.544 (2)	C18—H18	1.0000
C2—C5	1.563 (2)	C19—C20	1.518 (2)
C2—H2	1.0000	C20—C21	1.541 (2)
C3—C4	1.508 (2)	C20—H20	1.0000
C3—C6	1.567 (2)	C21—C22	1.504 (2)
C3—H3	1.0000	C21—H21	1.0000
C5—C8	1.525 (2)	C23—C24	1.523 (2)
C5—H5	1.0000	C23—H23A	0.9900
C6—C7	1.514 (2)	C23—H23B	0.9900
C6—H6	1.0000	C24—C25	1.518 (2)
C7—C8	1.376 (2)	C24—H24A	0.9900
C7—C16	1.406 (2)	C24—H24B	0.9900
C8—C9	1.415 (2)	C25—H25A	0.9900
C9—C10	1.420 (2)	C25—H25B	0.9900
C9—C14	1.445 (2)		
			
C5—O3—C6	96.84 (11)	C14—C13—C18	132.49 (14)
C18—O4—C17	96.65 (11)	C13—C14—C15	122.96 (14)
C1—N1—C4	113.15 (14)	C13—C14—C9	116.35 (14)
C1—N1—C25	122.21 (13)	C15—C14—C9	120.55 (14)
C4—N1—C25	123.88 (13)	C16—C15—C14	121.27 (15)
C22—N2—C19	113.30 (14)	C16—C15—H15	119.4
C22—N2—C23	122.69 (13)	C14—C15—H15	119.4
C19—N2—C23	122.87 (14)	C15—C16—C7	118.23 (14)
O1—C1—N1	123.81 (16)	C15—C16—H16	120.9
O1—C1—C2	127.59 (15)	C7—C16—H16	120.9
N1—C1—C2	108.52 (13)	O4—C17—C12	100.93 (13)
C1—C2—C3	105.10 (13)	O4—C17—C20	100.38 (13)
C1—C2—C5	114.57 (13)	C12—C17—C20	107.96 (13)
C3—C2—C5	101.47 (12)	O4—C17—H17	115.2
C1—C2—H2	111.7	C12—C17—H17	115.2
C3—C2—H2	111.7	C20—C17—H17	115.2
C5—C2—H2	111.7	O4—C18—C13	101.20 (12)
C4—C3—C2	104.59 (13)	O4—C18—C21	98.67 (12)
C4—C3—C6	114.28 (13)	C13—C18—C21	110.69 (13)
C2—C3—C6	101.62 (12)	O4—C18—H18	114.8
C4—C3—H3	111.9	C13—C18—H18	114.8
C2—C3—H3	111.9	C21—C18—H18	114.8
C6—C3—H3	111.9	O5—C19—N2	123.13 (16)
O2—C4—N1	123.67 (15)	O5—C19—C20	128.48 (15)
O2—C4—C3	127.84 (15)	N2—C19—C20	108.39 (14)
N1—C4—C3	108.46 (13)	C19—C20—C21	104.03 (13)
O3—C5—C8	101.09 (12)	C19—C20—C17	115.30 (14)
O3—C5—C2	99.32 (12)	C21—C20—C17	101.97 (13)
C8—C5—C2	109.09 (13)	C19—C20—H20	111.6
O3—C5—H5	115.1	C21—C20—H20	111.6
C8—C5—H5	115.1	C17—C20—H20	111.6
C2—C5—H5	115.1	C22—C21—C20	105.53 (14)
O3—C6—C7	101.01 (12)	C22—C21—C18	115.50 (13)
O3—C6—C3	100.58 (12)	C20—C21—C18	100.74 (13)
C7—C6—C3	107.61 (13)	C22—C21—H21	111.5
O3—C6—H6	115.3	C20—C21—H21	111.5
C7—C6—H6	115.3	C18—C21—H21	111.5
C3—C6—H6	115.3	O6—C22—N2	123.74 (15)
C8—C7—C16	122.16 (15)	O6—C22—C21	128.01 (16)
C8—C7—C6	105.20 (13)	N2—C22—C21	108.25 (14)
C16—C7—C6	132.46 (15)	N2—C23—C24	108.10 (13)
C7—C8—C9	121.47 (14)	N2—C23—H23A	110.1
C7—C8—C5	105.19 (13)	C24—C23—H23A	110.1
C9—C8—C5	133.02 (14)	N2—C23—H23B	110.1
C8—C9—C10	123.95 (15)	C24—C23—H23B	110.1
C8—C9—C14	116.05 (14)	H23A—C23—H23B	108.4
C10—C9—C14	119.89 (15)	C25—C24—C23	115.17 (13)
C11—C10—C9	121.64 (15)	C25—C24—H24A	108.5
C11—C10—H10	119.2	C23—C24—H24A	108.5
C9—C10—H10	119.2	C25—C24—H24B	108.5
C10—C11—C12	118.22 (15)	C23—C24—H24B	108.5
C10—C11—H11	120.9	H24A—C24—H24B	107.5
C12—C11—H11	120.9	N1—C25—C24	108.51 (13)
C13—C12—C11	121.82 (15)	N1—C25—H25A	110.0
C13—C12—C17	104.63 (14)	C24—C25—H25A	110.0
C11—C12—C17	133.45 (15)	N1—C25—H25B	110.0
C12—C13—C14	121.86 (15)	C24—C25—H25B	110.0
C12—C13—C18	105.53 (14)	H25A—C25—H25B	108.4
			
C4—N1—C1—O1	178.33 (15)	C18—C13—C14—C15	−4.6 (3)
C25—N1—C1—O1	7.9 (2)	C12—C13—C14—C9	−5.0 (2)
C4—N1—C1—C2	1.29 (18)	C18—C13—C14—C9	179.76 (15)
C25—N1—C1—C2	−169.11 (13)	C8—C9—C14—C13	178.42 (13)
O1—C1—C2—C3	179.59 (16)	C10—C9—C14—C13	2.1 (2)
N1—C1—C2—C3	−3.51 (16)	C8—C9—C14—C15	2.7 (2)
O1—C1—C2—C5	−69.9 (2)	C10—C9—C14—C15	−173.62 (14)
N1—C1—C2—C5	106.97 (15)	C13—C14—C15—C16	−173.38 (15)
C1—C2—C3—C4	4.25 (16)	C9—C14—C15—C16	2.0 (2)
C5—C2—C3—C4	−115.36 (13)	C14—C15—C16—C7	−4.3 (2)
C1—C2—C3—C6	123.42 (13)	C8—C7—C16—C15	1.8 (2)
C5—C2—C3—C6	3.80 (15)	C6—C7—C16—C15	176.25 (16)
C1—N1—C4—O2	−176.40 (14)	C18—O4—C17—C12	52.79 (14)
C25—N1—C4—O2	−6.2 (2)	C18—O4—C17—C20	−57.98 (13)
C1—N1—C4—C3	1.60 (18)	C13—C12—C17—O4	−35.42 (15)
C25—N1—C4—C3	171.81 (13)	C11—C12—C17—O4	148.25 (17)
C2—C3—C4—O2	174.23 (15)	C13—C12—C17—C20	69.39 (16)
C6—C3—C4—O2	64.0 (2)	C11—C12—C17—C20	−106.9 (2)
C2—C3—C4—N1	−3.66 (16)	C17—O4—C18—C13	−50.62 (13)
C6—C3—C4—N1	−113.89 (14)	C17—O4—C18—C21	62.61 (13)
C6—O3—C5—C8	−50.90 (13)	C12—C13—C18—O4	30.01 (15)
C6—O3—C5—C2	60.80 (13)	C14—C13—C18—O4	−154.14 (16)
C1—C2—C5—O3	−151.62 (13)	C12—C13—C18—C21	−73.82 (16)
C3—C2—C5—O3	−38.98 (14)	C14—C13—C18—C21	102.04 (19)
C1—C2—C5—C8	−46.38 (18)	C22—N2—C19—O5	−177.28 (16)
C3—C2—C5—C8	66.26 (15)	C23—N2—C19—O5	−9.2 (3)
C5—O3—C6—C7	52.06 (13)	C22—N2—C19—C20	2.06 (18)
C5—O3—C6—C3	−58.41 (13)	C23—N2—C19—C20	170.12 (14)
C4—C3—C6—O3	144.55 (13)	O5—C19—C20—C21	173.59 (18)
C2—C3—C6—O3	32.53 (14)	N2—C19—C20—C21	−5.71 (17)
C4—C3—C6—C7	39.30 (18)	O5—C19—C20—C17	62.8 (2)
C2—C3—C6—C7	−72.71 (15)	N2—C19—C20—C17	−116.47 (15)
O3—C6—C7—C8	−33.94 (15)	O4—C17—C20—C19	142.32 (13)
C3—C6—C7—C8	71.00 (15)	C12—C17—C20—C19	37.14 (18)
O3—C6—C7—C16	150.96 (16)	O4—C17—C20—C21	30.35 (14)
C3—C6—C7—C16	−104.09 (18)	C12—C17—C20—C21	−74.84 (15)
C16—C7—C8—C9	3.1 (2)	C19—C20—C21—C22	7.03 (17)
C6—C7—C8—C9	−172.63 (14)	C17—C20—C21—C22	127.24 (13)
C16—C7—C8—C5	177.36 (13)	C19—C20—C21—C18	−113.47 (14)
C6—C7—C8—C5	1.63 (16)	C17—C20—C21—C18	6.74 (15)
O3—C5—C8—C7	31.17 (15)	O4—C18—C21—C22	−154.94 (13)
C2—C5—C8—C7	−72.85 (16)	C13—C18—C21—C22	−49.42 (18)
O3—C5—C8—C9	−155.52 (16)	O4—C18—C21—C20	−41.83 (14)
C2—C5—C8—C9	100.45 (19)	C13—C18—C21—C20	63.69 (15)
C7—C8—C9—C10	170.97 (14)	C19—N2—C22—O6	−178.31 (15)
C5—C8—C9—C10	−1.5 (3)	C23—N2—C22—O6	13.6 (2)
C7—C8—C9—C14	−5.2 (2)	C19—N2—C22—C21	2.70 (19)
C5—C8—C9—C14	−177.62 (15)	C23—N2—C22—C21	−165.38 (13)
C8—C9—C10—C11	−173.83 (15)	C20—C21—C22—O6	174.90 (16)
C14—C9—C10—C11	2.2 (2)	C18—C21—C22—O6	−74.8 (2)
C9—C10—C11—C12	−3.7 (2)	C20—C21—C22—N2	−6.16 (17)
C10—C11—C12—C13	0.8 (2)	C18—C21—C22—N2	104.13 (16)
C10—C11—C12—C17	176.65 (16)	C22—N2—C23—C24	76.48 (19)
C11—C12—C13—C14	3.6 (2)	C19—N2—C23—C24	−90.46 (18)
C17—C12—C13—C14	−173.23 (14)	N2—C23—C24—C25	178.61 (14)
C11—C12—C13—C18	−179.96 (14)	C1—N1—C25—C24	76.11 (19)
C17—C12—C13—C18	3.17 (16)	C4—N1—C25—C24	−93.24 (17)
C12—C13—C14—C15	170.65 (15)	C23—C24—C25—N1	−179.18 (14)
